# Acute on chronic bilateral renal vein thrombosis in the setting of remission of class V lupus nephritis: A case report and literature review 

**DOI:** 10.5414/CNCS110922

**Published:** 2023-03-05

**Authors:** Christopher El Mouhayyar, Christine Segal, Bertrand L. Jaber, Vaidyanathapuram S. Balakrishnan

**Affiliations:** 1 Department of Medicine, Division of Nephrology, St. Elizabeth’s Medical Center,; 2 Department of Medicine, Tufts University School of Medicine, and; 3 Department of Radiology, St. Elizabeth’s Medical Center, Boston, MA, USA

**Keywords:** acute, chronic, renal vein thrombosis, lupus nephritis, membranous nephropathy

## Abstract

Renal vein thrombosis (RVT), defined as the presence of a thrombus in the major renal vein or one of its tributaries, can present acutely or go unnoticed resulting in acute kidney injury or chronic kidney disease. RVT is associated with multiple etiologies, including nephrotic syndrome, thrombophilia, autoimmune disorders, and malignancy. Patients with systemic lupus erythematosus (SLE), a multiorgan autoimmune disorder, are predisposed to coagulopathy and thus are at a higher risk of venous and arterial thromboembolism. We describe the case of a 41-year-old man with SLE and biopsy-proven membranous glomerulonephritis (WHO class V lupus nephritis) in clinical remission with no evidence of nephrotic range proteinuria who presented with macroscopic hematuria and was diagnosed with acute-on-chronic bilateral RVT. We discuss the different causes of RVT and compare the clinical presentation, diagnostic imaging findings, and management of acute and chronic RVT.

## Introduction 

Renal vein thrombosis (RVT), the presence of a thrombus in the major renal vein or one of its tributaries, can present acutely or go unnoticed due to lack of symptoms resulting in acute kidney injury or chronic kidney disease. In the 1840s, Rayer, a French nephrologist, was the first to describe RVT and its association with proteinuria [1]. RVT is more commonly seen in men with no racial predilection, and almost two third present with bilateral renal vein involvement [1]. RVT is associated with multiple etiologies, including nephrotic syndrome, thrombophilia, autoimmune disorders, and malignancy. Systemic lupus erythematosus (SLE) is a multiorgan autoimmune disorder characterized by a broad spectrum of clinical manifestations and serological findings. Patients with SLE are predisposed to coagulopathy and thus are at a higher risk of venous and arterial thromboembolism [2]. 

We present the case of a 41-year-old man with SLE and biopsy-proven membranous glomerulonephritis (World Health Organization (WHO) class V lupus nephritis) in clinical remission with no evidence of nephrotic range proteinuria presenting with macroscopic hematuria and found to have bilateral acute-on-chronic RVT. 

## Case presentation 

A 41-year-old man with SLE and biopsy-proven membranous glomerulonephritis (WHO class V lupus nephritis) diagnosed 2.5 years earlier, maintained on mycophenolate mofetil (1,000 mg twice daily), prednisone (5 mg daily), and hydroxychloroquine (200 mg daily), hypertension, and hypothyroidism presented to the hospital with bilateral flank pain of 1 month duration, gross hematuria for 2 days, and nausea and vomiting for 1 day. He was afebrile with a normal heart rate of 71 beats/minute. He was hypertensive with a blood pressure of 172/110 mmHg and oxygen saturation was 100% on room air. On physical examination, there was mild diffuse abdominal tenderness but no costovertebral angle tenderness, and trace lower extremity edema. At initial presentation, the white blood cell count was elevated at 13,000/μL. C-reactive protein level was elevated at 0.81 mg/dL (normal level, < 0.5), and LDH was elevated at 313 U/L (range, 102 – 266). Initial serum creatinine was 1.4 mg/dL with serum potassium of 2.9 mEq/L and serum albumin of 4.4 g/dL. Random urine total protein-to-creatinine ratio was 300 mg/g and random urine albumin-to-creatinine ratio was 81 mg/g. Of note, 9 months prior to presentation, serum creatinine was 1.1 mg/dL with an estimated glomerular filtration rate (GFR) of 83 mL/minute/1.73m^2^, and random urine total protein-to-creatinine ratio was 691 mg/g. A nasal swab SARS-CoV-2 nucleic acid amplification test was negative. His lipid profile was unremarkable, with a total cholesterol of 95 mg/dL and an LDL cholesterol of 42 mg/dL. Non-contrast computed tomography (CT) scan revealed enlarged kidneys bilaterally, more prominent on the right, with bilateral perinephric edema. There was significant narrowing of the renal veins bilaterally, and numerous venous collaterals in the perinephric space suggestive of bilateral RVT. Increased density in the left renal pelvis and bladder were suggestive of hemorrhage within the collecting system. He was initiated on systemic anticoagulation with a continuous infusion of unfractionated heparin, and a Foley catheter was inserted with continuous bladder irrigation. 

The patient developed acute kidney injury, and his serum creatinine peaked at 1.9 mg/dL in the first 48 hours of hospitalization. An extensive workup for thrombophilia was undertaken and was inconclusive. Prothrombin time, partial thromboplastin time, and fibrinogen level were normal. Functional protein S was 86% (normal range, 63 – 140%), and functional protein C was 72% (normal range, 73 – 180%). Anti-thrombin III activity was 86% (normal range, 75 – 135%). Factor V Leiden mutation as well as prothrombin G20210A mutation were absent. Serum homocysteine level was mildly elevated at 15.2 μmol/L (normal range, < 13.0). Antinuclear antibodies screening test was positive, but the anti-double-stranded DNA antibody titer was undetectable. Levels of complement components C3 and C4 were normal. Rheumatoid factor and anti-Smith antibody titer were undetected. Antineutrophil cytoplasmic antibody titers, including anti-myeloperoxidase and anti-proteinase-3 antibody titers, were undetected. The anti-RNP (ribonucleoproteins) antibody titer was elevated at 3.9 AI (normal value, < 0.9). Anti-phospholipid autoantibodies were undetectable, specifically, anti-cardiolipin IgA, IgG, and IgM antibody titers, and anti-β-2 glycoprotein-I IgA, IgG, and IgM antibody titers. Lupus anticoagulant screen was negative. 

**Figure 1.**
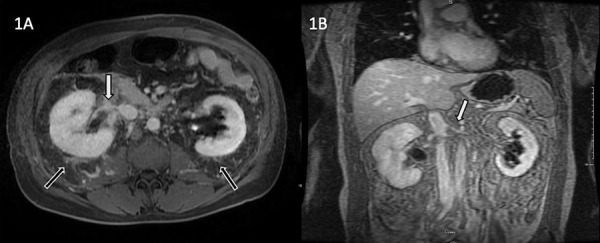
Magnetic resonance venography of the kidneys demonstrating marked narrowing and poor opacification of the renal veins bilaterally with perinephric venous collaterals. A: Axial T1 fat-saturated image obtained 5 minutes following contrast administration demonstrates diffuse narrowing and irregularity of the right renal vein (white arrow) and the presence of bilateral perinephric collateral veins (black arrow). B: Coronal volumetric interpolated breath-hold examination (VIBE) image obtained 6 minutes following contrast administration demonstrates narrowing and irregularity of the distal left renal vein (white arrow).

**Figure 2.**
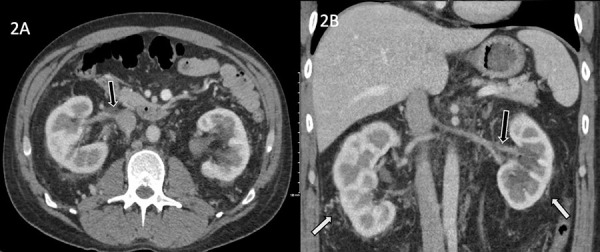
Computed tomography angiography of the kidneys demonstrating bilateral renal vein thrombosis and perinephric venous collaterals. A: Axial contrast-enhanced image demonstrates a filling defect in the distal right renal vein (black arrow) and bilateral perinephric collateral veins. There is left-sided hydronephrosis with increased density in the left renal pelvis likely representing hemorrhage. B: Coronal contrast-enhanced image demonstrates a filling defect within the proximal left renal vein (black arrow) and bilateral perinephric collateral veins (white arrow).

Magnetic resonance venography (MRV) (Figure 1) revealed markedly narrowed bilateral renal veins with poor contrast opacification. No obvious acute RVT was identified, and the appearance of the study suggested a more chronic process, possibly with chronic thrombosis, or scarring from chronic thrombosis, particularly due to the extent of perinephric collateral veins. A CT venogram was suggested to improve the spatial resolution provided by CT and to evaluate for acute thrombus. After pre-hydration with 1 L of normal saline, the patient underwent a contrast-enhanced CT of the abdomen (Figure 2), which demonstrated filling defects in the renal veins bilaterally consistent with acute thrombus and marked attenuation of the distal left renal vein. 

During hospitalization, the kidney function progressively normalized with a drop in serum creatinine to 0.95 mg/dL, and hematuria resolved. The patient was discharged on warfarin therapy (with an international normalized ratio (INR) target of 2 – 3) and his immunosuppressive medications. Three months following hospital discharge, the patient had no recurrence of gross hematuria, serum creatinine was normal at 0.95 mg/dL, complement levels remained normal, and anti-double-stranded DNA antibody titer undetected. 

## Discussion 

RVT can present acutely or can be chronic in nature. Acute RVT usually presents with symptoms of flank pain and tenderness, worsening kidney function, proteinuria and hematuria, and is most often due to a hypercoagulable state [2]. By contrast, chronic RVT is usually asymptomatic or insidious in onset, and may rarely present with peripheral edema or be identified during a workup for another culprit such as pulmonary embolism [2, 3]. Interestingly, our patient’s symptoms were acute as he had flank pain for almost a month and gross hematuria for 2 days. However, the imaging findings revealed bilateral renal veins of small caliber with perinephric collateral veins suggesting chronicity and raising the possibility of acute-on-chronic RVT (Table 1). 

Hypercoagulability, commonly seen with active nephrotic syndrome, especially membranous glomerulonephritis, typically with serum albumin less than 2.8 gm/dL, is associated with a higher risk of RVT [4]. It appears to be related to urinary loss of anti-coagulation factors such as antithrombin III, plasminogen, and protein C and S. Our patient did not have proteinuria, his serum albumin was normal at 4.4 gm/dL, and activity of these coagulation factors was within the normal range. Other common causes of hypercoagulability include factor V Leiden mutation, prothrombin G20210A mutation as well as antiphospholipid syndrome. However, our patient’s anti-cardiolipin antibody and anti-β-2 glycoprotein-I antibody titers were undetectable, and the lupus anticoagulant screen was negative. He also did not carry the factor V Leiden or prothrombin G20210A mutation. 

Dyslipidemia plays a role in accelerating atherosclerosis and thus might have a causative role in the increased risk of thrombosis seen in nephrotic syndrome [5]. However, our patient’s lipid profile was within normal limits making dyslipidemia less likely an inciting factor. 

Renal malignancy, especially renal cell carcinoma that extends into the renal veins, is also associated with RVT most likely by extrinsic compression of the renal vein [6]. This can be also seen with enlarged retroperitoneal lymphadenopathy, an abdominal aortic aneurysm, or a retroperitoneal tumor compressing the renal vein or inferior vena cava. However, abdominal imaging in our patient did not show any evidence of malignancy, aortic aneurysm, or lymphadenopathy making this less likely. 

Renal vein thrombosis in the setting of lupus nephritis is more commonly associated with lupus membranous glomerulonephritis as well as the antiphospholipid syndrome [7]. Anti-double-stranded DNA antibody titers and complement levels closely correlate with disease activity in lupus nephritis [8, 9]. Our patient’s complement levels were normal, anti-double-stranded DNA antibody titer undetectable, lupus membranous glomerulonephritis well controlled on immunosuppressive medications with no active flare on presentation, and his nephrotic syndrome was in remission. 

Although anti-RNP antibodies are detectable in 25 – 40% of patients with SLE [10], these autoantibodies are not specific to SLE and can be found in other rheumatological disorders. High titers of anti-RNP antibodies are considered as a classification criterion for mixed connective tissue disease (MCTD) with a high sensitivity but low specificity [10]. Unlike other autoantibodies, anti-RNP antibodies do not correlate with disease activity. Our patient had a mildly elevated anti-RNP antibody titer, which is of unclear significance, as he did not have any other presenting features that meet the criteria for MCTD. 

In conclusion, RVT is caused by a multitude of conditions and can present either acutely or go unnoticed thus inducing acute or chronic kidney disease. In this case report, we present a patient with acute-on-chronic RVT and discuss the clinical presentation, diagnosis, and treatment modalities. 

## Funding 

The authors were not the recipients of any internal or external funding for this work. 

## Conflict of interest 

The authors declare no conflict of interest or funding pertaining to this manuscript. 

**Table 1.** Comparison of acute versus chronic renal vein thrombosis.

	Acute renal vein thrombosis	Chronic renal vein thrombosis
Clinical presentation	Acute flank pain, tenderness, nausea, vomiting Microscopic or macroscopic hematuria, proteinuria Worsening kidney function	Insidious, asymptomatic Lower extremity edema Worsening kidney function
Diagnosis	Imaging study of choice: Computed tomography angiography (sensitivity and specificity of almost 100% [11]), or magnetic resonance venography Gold standard diagnostic imaging study: Renal venography (has the additional advantage of potentially being a therapeutic procedure; rarely used in clinical practice due to the availability of less invasive imaging studies)	
Imaging findings	Enlarged kidneys with persistent cortical enhancement Changes in attenuation, either focal or diffuse, may be present due to perfusion abnormalities	Presence of perinephric collateral veins, suggestive of chronicity
Treatment	Treatment of the underlying disorder Unfractionated or low-molecular-weight heparin with bridge to warfarin therapy (international normalized ratio target of 2 – 3) Catheter-based mechanical/pharmacological thrombectomy and/or systemic pharmacological thrombolysis Inferior vena cava filter placement if contraindication to anticoagulation (for prevention of pulmonary embolism)	
